# A Novel Model to Predict Inadequate Bowel Preparation Prior to Colonoscopy Incorporating Patients’ Reactions to Drinking the Laxative

**DOI:** 10.3390/jcm12237335

**Published:** 2023-11-26

**Authors:** Daniela Malkin, Daniel L. Cohen, Vered Richter, Eran Ariam, Sergei Vosko, Haim Shirin, Anton Bermont

**Affiliations:** The Gonczarowski Family Institute of Gastroenterology and Liver Diseases, Shamir (Assaf Harofeh) Medical Center, Zerifin 70300, Israel; danielalevy7@hotmail.com (D.M.); danielc@shamir.gov.il (D.L.C.); veredr@shamir.gov.il (V.R.); eranar@shamir.gov.il (E.A.); sergeivo@shamir.gov.il (S.V.); haimsh@shamir.gov.il (H.S.)

**Keywords:** colonoscopy, bowel preparation, inadequate preparation

## Abstract

Background and Aims: Prior studies have identified predictors of inadequate preparation with limited success. We aimed to build a model that could predict the likelihood of inadequate preparation by also including factors related to the patient’s reaction to drinking the laxative preparation. Methods: Demographic, clinical, and preparation-related data were prospectively collected on patients undergoing colonoscopy. An inadequate preparation was defined as a Boston Bowel Preparation Scale < 6. Statistical analyses were performed to identify predictors of inadequate preparation and create a predictive model. Results: 324 patients were included (age 67 +/− 14 years, 52% male). 77 (23.7%) had inadequate preparations. Diabetes (*p* < 0.001), cerebrovascular accident (CVA) (*p* < 0.001), incomplete prep consumption (*p* = 0.007), high school level education and above (*p* < 0.001), use of Bisacodyl (*p* = 0.005), >10 bowel movements (*p* = 0.02), and use of Sodium Picosulfate or low-volume polyethylene glycol (PEG) solution (2L) compared to PEG 3L (*p* < 0.001) were significant variables. In a multivariate analysis, prior CVA increased the risk for inadequate preparation (OR = 4.8, CI 1.6–14.5), whereas high school level education and above (OR = 0.4, CI 0.2–0.8), consumption of Bisacodyl (OR = 0.4, CI 0.2–0.8), >10 bowel movements (OR = 0.5, CI 0.3–0.9), and use of Sodium Picosulfate (OR = 0.5, CI 0.3–0.9) decreased the risk for inadequate prep. Using these, a predictive model for patients likely to have an inadequate colon preparation was created with an area under the curve of 0.74 (35% sensitivity, 90% specificity at a cutoff point of 39%). Conclusion: Given the low sensitivity, this predictive model does not appear ready for clinical use. However, due to its high specificity, it may be helpful in high-risk, sicker populations by preventing inadequately prepped procedures.

## 1. Introduction

Colonoscopy is the gold standard for detecting conditions such as colon polyps, cancer, inflammation, and angioectasias. An adequate level of bowel preparation is essential for a successful procedure. This decreases the risk of missed lesions and prevents the need for repeat testing and additional labor, thereby reducing cost [1,2]. Despite this, approximately 20–30% of patients who undergo colonoscopy are not adequately prepared [3,4], leading to difficult and prolonged procedures, higher complication rates, and the risk of missed pathological lesions [5].

Patients who are hospitalized or undergoing direct-access colonoscopy may not receive preparation instructions from a gastroenterologist and may be at a higher risk of inadequate preparation. Thus, it is important that physicians of different specialties be knowledgeable concerning colonoscopy preparation regimens and risk factors for inadequate preparation.

Several scales are used to assess the quality of bowel preparation, including the Aronchick Scale, Boston Bowel Preparation Scale (BBPS), and the Ottawa Bowel Preparation Scale. These scales rely on subjective physician assessment [6].

Most prior research has examined the risk factors and predictors of an inadequate colon prep with the goal of identifying patients who may require a more intensive bowel cleansing prior to colonoscopy [7,8]. In a number of studies, researchers have been successful in building models able to predict about 50–60% of those patients likely to be inadequately prepped [9,10,11]. Factors known to be associated with inadequate preparation include obesity, male sex, advanced age, cirrhosis, Parkinson’s disease, diabetes, chronic constipation, and a prior inadequate preparation. Surprisingly, these predictors are all related to a patient’s medical history, and do not include factors related to the patients’ compliance with the laxative preparation or a patient’s response to it.

One recent study examined whether the color and consistency of patients’ feces during prep was a predictive factor for the cleanliness of the colon. They found that patients who reported that their last bowel movement prior to the procedure was liquid brown or firm were more likely to be inadequately prepared [12].

Given the lack of prior studies assessing this, we aimed to evaluate predictors of an inadequate bowel preparation, including factors related to a patient’s compliance with the laxative regimen and the consistency of their bowel movements after drinking the preparation. Further, after identifying the predictors of an inadequate preparation, we sought to create a highly accurate predictive model incorporating these factors to identify patients likely to have an inadequate preparation.

## 2. Materials and Methods

### 2.1. Study Design

A prospective real-world study was conducted at a public tertiary-referral medical center between October 2021 and June 2022. All patients undergoing elective colonoscopy and who were able to fill out the patient questionnaire were invited to participate. This included both inpatients and outpatients, and did not depend upon the indication for the procedure. Before the colonoscopy, the patients received a questionnaire that included demographic and clinical data, as well as social habits, adherence to prep instructions, and reaction to the prep. Constipation was defined as less than three bowel movements per week. Bowel prep regimens included Sodium Picosulfate (Pico Salax, Ferring GmbH, Kiel, Germany), low-volume polyethylene glycol (PEG) solution (2 L) (Moviprep, Norgine Limited, Uxbridge, UK), and PEG 3 L (Meroken, Taro Pharmaceutical Industries LTD, Haifa, Israel). The laxative regimen used was based on the referring physician’s choice, and was therefore not randomized. In addition to those laxative regimens, additional laxatives such as Bisacodyl may have been prescribed per the referring physician’s discretion.

After completing the questionnaire, the subjects then underwent colonoscopy. The colon cleanliness was graded by the performing endoscopist according to the BPPS, with a total score < 6 considered to be inadequately prepared. All colonoscopies were performed by experienced attending gastroenterologists or fellows under the supervision of an attending gastroenterologist.

Patients were ineligible for the study if they were undergoing an urgent colonoscopy, were unable to fill out the questionnaire, or refused to participate. By filling out the questionnaire, all participants provided informed consent. The study was approved by the medical center’s Institutional Review Board (approval #ASF-0160-21).

### 2.2. Statistical Analysis

Categorical variables were summarized as frequency and percentage. Continuous variables were evaluated for normal distribution using a histogram. Since all continuous variables were skewed, they were reported as median and interquartile range. Chi Square Test and Fischer Exact Test were applied to compare categorical variables between those with and without and adequate prep, while Mann–Whitney Test was used to compare continuous variables.

Variables that were significantly associated with bowel cleanliness were included in a multivariate analysis. Backward likelihood ratio multivariate logistic regression analysis was used to identify adequate bowel preparation predictors and build the prediction model (*p* > 0.1 for removal from the model). The area under the receiver operating characteristic curve allows discrimination between patients with and without adequate bowel preparation. The Hosmer–Lemeshow goodness-of-fit test was used to evaluate the calibration. We looked for a model probability threshold that would give 90% specificity as the model was directed to achieve a low false positive rate.

All statistical tests were 2-sided and *p* < 0.05 was considered statistically significant. SPSS was used for all statistical analysis (IBM SPSS Statistics for Windows, version 28, IBM Corporation, Armonk, New York, NY, USA, 2021).

## 3. Results

### 3.1. Study Population

A total of 324 subjects were included in the study (see Table 1). The average age of the subjects was 67 +/− 14 years old and 52% were male. Overall, the mean BMI was 26.9, 29.9% had diabetes, and 48.9% had greater than a high school level of education.

### 3.2. Variables Associated with an Inadequate Bowel Preparation

Of the cohort, 77 of the patients (23.8%) had inadequate bowel preparation. Factors predisposing towards an inadequate prep included diabetes (*p* < 0.001), a prior cerebrovascular accident (CVA) (*p* < 0.001), and partial consumption of the preparation solution (*p* = 0.007). Factors significantly associated with a good preparation included greater than a high school level of education (*p* < 0.001), preparation with Sodium Picosulfate or low-volume PEG compared to PEG 3L (*p* = 0.002), Bisacodyl use 48 h prior to the colonoscopy (*p* = 0.005), and more than 10 bowel movements during preparation (*p* = 0.02) (see Table 1).

Other factors, including the indication for colonoscopy or history of a prior abdominal surgery, did not significantly affect the bowel cleanliness. Furthermore, use of Bisacodyl 24 h prior to colonoscopy was not found to be statistically significant, nor were the use of other medications and nutritional supplements. Finally, there was no difference in prep cleanliness based on the different methods in which the preparation instructions were provided to the patient, such as written instructions only versus written and oral instructions.

### 3.3. Multivariate Logistic Regression Analysis of Variables Associated with an Inadequate Bowel Preparation

Stepwise backward logistic regression analysis (Table 2) revealed that prior CVA increased the risk for an inadequate preparation (OR = 4.9, 95% CI 1.6 -14.5), whereas high school level of education and above (OR = 0.4, 95% CI 0.2–0.8), Bisacodyl use 48 h prior to the colonoscopy (OR = 0.4, 95% CI 0.2–0.8), more than ten bowel movements during prep (OR = 0.5, 95% CI 0.2–0.9), consumption of Sodium Picosulfate compared to PEG 3L (OR = 0.5, 95%. CI 0.3–0.9), or consumption of low-volume polyethylene glycol (PEG) solution compared to PEG 3L (OR = 0.2, 95% CI 0.07–0.7) all decreased the risk of having an inadequate preparation.

### 3.4. Creating a Model to Predict Inadequate Bowel Preparation

Based on the results of the multivariate regression analysis, a model was built to predict inadequate bowel preparation. The probability of having inadequate preparation. was calculated by using the following equation:

PIP = 1/(1 + e^−Z^)

where PIP is the probability of having inadequate preparation, e is 2.718 and

Z = 0.735 − 0.923 × GE − 0.723 × BPRP − 1.511 × BPR2P + 1.584 × CVA − 0.678 × BM − 0.806 × B

where GE is grade of education (if high school level of education or higher: −1; if less: 0); BPRP is bowel preparation regimen (if Sodium Picosulfate was used: −1, if no: 0); BPR2P is bowel preparation regimen with low-volume PEG solution (if used: −1, if not: 0); CVA (if yes: −1, if no: 0); BM is bowel movement above 10 (if yes: −1, if no: 0); and B is Bisacodyl 48 h prior (if yes: 1, if no: 0).

**Figure 1:**
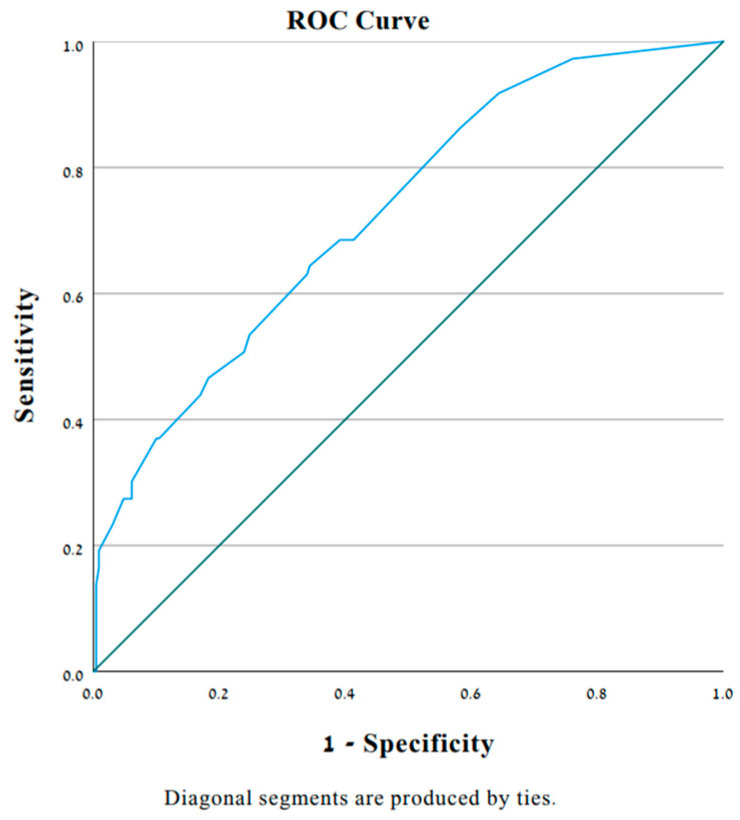
The area under the receiver operating characteristic curve ROC. Used to evaluate the discrimination ability of our predictive model.

This logistic model showed good discrimination and calibration ability with an area under the curve of 0.737 [95% CI: 0.673, 0.8] (Figure 1); Hosmer–Lemeshow goodness-of-fit test, x^2^ = 8.399; *p* = 0.299. Receiver operating characteristic analysis allowed the identification that a threshold value of 0.39 provided 35% sensitivity and 90% specificity.

## 4. Discussion

This study attempted to build a highly predictive model that would reliably predict those with inadequate bowel cleansing just prior to the procedure. In contrast to previous studies, we attempted to add information related to how the patients drank and reacted to the laxative preparation to increase the accuracy of our model. However, despite our effort and a high specificity, our model suffered from a low sensitivity.

Many colonoscopies still have inadequate preparations, and poor preparations have been shown to affect the outcome of the procedure. A large multicenter study throughout Europe found that poor preps were associated with a higher percentage of incomplete procedures, longer intubation and withdrawal times, a need for fluoroscopy assistance, and a more difficult procedure [5]. Further, the polyp detection rate decreased. Other studies have shown that patients with a poor preparation are at increased risk of perforation [13]. Thus, a model to predict inadequate preparation prior to the examination may prevent unsuccessful colonoscopies, missed lesions, patient complications, and save time and money.

The risk factors for inadequate bowel preparation have been assessed in multiple studies. For example, Ness et al. identified a later colonoscopy starting time, failure to follow prep instructions, inpatient status, constipation, male gender, tricyclic antidepressant use, cirrhosis, stroke and dementia as predictors of an inadequate preparation [8]. More recently, Zad et al. identified additional factors, including male gender, diabetes and a history of prior abdominal surgery [7]. A systematic review and meta-analysis on this subject including nearly 50,000 subjects from 24 studies concluded that the following factors predicted an inadequate preparation: older age, male gender, inpatient status, diabetes, hypertension, cirrhosis, narcotic use, constipation, stroke and tricyclic antidepressant use [14].

Based on these variables, several prior studies have attempted to build a model that would predict which patients are at high-risk of having an inadequate preparation and therefore require a more aggressive laxative regimen. One such study was conducted by Hassan et al., in which they devised a clinical model to predict 60% of patients with an inadequate prep. Their model included male sex, overweight, high BMI, older age, previous colorectal surgery, cirrhosis, Parkinson’s disease, diabetes and a positive fecal occult blood test. Including these variables, their model had a 60% sensitivity and 59% specificity [9]. Other subsequent predictive models have also been plagues by low sensitivity and/or specificity. For example, the model of Dik et al. [10]. showed a 43% sensitivity and 90% specificity; Gimeno-Garcia et al. showed 60.3% sensitivity and 75.4% specificity [11]; and Afecto et al. showed 60.3% sensitivity and 64.2% specificity [15].

In an attempt to build a better model, we sought to include patient descriptions of their reaction to the laxative preparation in the prediction model as this has not previously been incorporated. We identified predictors of an inadequate preparation (Table 2) and used these to build a predictive model. Of note, only one of the variables incorporated into the model was related to the patient’s response to the laxative (>10 bowel movements). Despite our efforts, the model suffered from a 35% sensitivity although the specificity (90%) was high.

Our study supplied additional data regarding the risk factors for inadequate bowel preparation. In a multivariate analysis, prior stroke and low education levels were found to have a significant negative effect on adequate bowel preparation, whereas taking Sodium Picosulfate or low-volume PEG, Bisacodyl 48 h prior to the colonoscopy, or having 10 or more bowel movements during the prep were found to be significant predictors of an adequate bowel preparation. Similar to our results, a study that compared preparation with PEG vs. Sodium Picosulfate found that Sodium Picosulfate was tolerated better, had fewer complications, and led to a cleaner colon [16]. Additionally, a study that compared the use of Sodium Picosulfate alone compared to Sodium Picosulfate together with Bisacodyl showed that the latter was superior for bowel preparation [17]. Moreover, patients suffering from chronic constipation benefited from the addition of Bisacodyl to their regimen [18]. Despite the abovementioned, the literature does not support the addition of Bisacodyl to prep regimens and various studies have not shown major differences in bowel prep with or without Bisacodyl. Moreover, it was shown that Bisacodyl led to more side effects, such as bloating, cramping and, in rare cases, to ischemic colitis [19].

This study has several limitations. First, it was performed at a single institution and had a relatively low number of subjects. The smaller number of participants may have limited our ability to identify all significant predictors of an inadequate preparation. It may also explain some of the differences in variables that were significant in other studies but not ours. There may have been other limitations given the study design such as patient reliability in fully answering the questionnaire and incomplete recall regarding medication use. By using real world patients, we were not able to randomize them to different preparation regimens and thus there were three groups. Additionally, the use of extra laxatives such as Bisacodyl, which had previously been recommended by the Israeli Gastroenterology Association but is no longer, was not controlled for and up to the discretion of the referring physician. Our subjects were also almost all outpatients who are generally a healthier population. Additionally, the BMI of our patients was nearly normal and the severity of diabetes was not addressed. Another issue is the lack of a validation group. While we constructed a model, we did not attempt to validate it by using another cohort of patients undergoing colonoscopy. Finally, although we included patient reactions to drinking the laxative preparation in our model, there is no data that this information is useful. In fact, some studies have suggested that patients are not reliable judges of the quality of their own bowel preparations [12,20].

In conclusion, unfortunately, given the low sensitivity, this model to predict inadequate bowel preparation prior to colonoscopy does not appear ready for universal use at this time. However, given to its high specificity, it may be beneficial for identifying which sicker patients, who are at high risk for complications during colonoscopy, are unlikely to be adequately prepped and should have additional laxatives given. A future study including a larger cohort of patients and with a modified questionnaire including additional information such as diabetes severity (HbA1c levels), social habits (alcohol consumption, physical activity), and added data on the reaction to the prep (estimated finish time of the prep, stool color, stool consistency) may improve such a model.

## Figures and Tables

**Table 1:** Baseline characteristics and preparation-related variables.

Baseline Characteristics and Prep Related Variables	N Patients	Boston Score < 6	Boston Score ≥ 6	*p*-Value
Overall	324	77 (23.8%)	247 (76.2%)	
Age (median [IQR])		67 (60–70)	64 (56–68)	0.16
Gender (Male)	169/324	45 (26.6%)	124 (73.4%)	
BMI (median [IQR])	319	27.5 [23.3–26.8]	27.9 [24.4–26.8]	0.69
Greater than high school level of education	158/323	29 (18.4%)	129 (81.6%)	<0.001
Comorbidities				
Diabetes	91/304	34 (37.4%)	57 (62.6%)	<0.001
Cerebrovascular accident	18/322	11 (61.1%)	7 (38.9%)	<0.001
Parkinson’s disease	7/322	3 (42.9%)	4 (57.1%)	0.34
Hypothyroidism	31/322	7 (22.6%)	24 (77.4%)	0.8
Prior abdominal surgery	159/322	40 (25.2%)	119 (74.8%)	0.6
Constipation	21/319	7 (33.3%)	14 (66.7%)	0.3
Medication use				
Neurally active medications	166/316	43 (25.9%)	123 (74.1%)	0.4
Anti-acid medications	115/316	33(28.7%)	82 (71.3%)	0.14
Anticholinergics	18/316	4 (22.2%)	14 (77.8%)	1
Fiber supplements	13/322	5 (38.5%)	8 (61.5%)	0.2
Chronic laxative use	35/324	10 (28.6%)	25 (71.4%)	0.48
Previous colonoscopy	318			0.71
First colonoscopy	79/318	16 (20.3%)	63 (79.7%)	
Good cleanliness	139/318	27 (19.4%)	112 (80.6%)	
Adequate cleanliness	18/318	3 (16.7%)	15 (83.3%)	
Poor cleanliness	23/318	8 (34.8%)	15 (65.2%)	
I do not know	59/318	38 (64.4%)	21 (35.6%)	
Bowel prep regimen				0.002
Polyethylene Glycol 3L	171/324	54 (31.6%)	117 (68.4%)	
Sodium Picosulfate	116/324	18 (15.5%)	98 (84.5%)	
low-volume PEG	37/324	5 (13.5%)	32 (86.5%)	
# hours fasting (median [IQR])	243	25.7 [19.2–24]	26 (24,24)	0.8
Prep not completely consumed	36/324	15 (41.7%)	21 (58.3%)	0.007
Bisacodyl 24 h prior to procedure	284/322	65 (23%)	219 (77%)	0.23
Bisacodyl 48 h prior to procedure	227/321	44 (19.4%)	183 (80.6%)	0.005
10 or more BM during prep	196/307	39 (19.9%)	157 (80.1%)	0.02
Clear bowels at end of prep	262/308	60 (22.9%)	202 (77.1%)	0.74

**Table 2:** Multivariable logistic regression analysis.

Variable	B	OR	95% CI	*p*-Value
High school level of education or higher	−0.923	0.4	0.2, 0.8	0.005
Bowel prep regimen				
Picosulfate vs. Polyethylene Glycol 3 L	−0.723	0.5	0.3, 0.9	0.028
Low-volume PEG vs. Polyethylene Glycol 3 L	−1.511	0.2	0.07, 0.7	0.013
Cerebrovascular accident	1.584	4.9	1.6, 14.5	0.005
10 or more bowel movements during preparation	−0.678	0.5	0.2, 0.9	0.024
Bisacodyl 48 h prior to procedure	−0.806	0.4	0.2, 0.8	0.008
Constant	0.735			

B—estimated coefficient.

## Data Availability

The research data is not publicly available due to privacy or ethical consideration. Once this manuscript is accepted for publication, delinked data without personal privacy could be provided upon reasonable request.
